# Retraction Note: Neurocognitive impairment and patient–proxy agreement on health-related quality of life evaluations in recurrent high-grade glioma patients

**DOI:** 10.1007/s11136-024-03696-y

**Published:** 2024-05-31

**Authors:** Ivan Caramanna, Martin Klein, Martin van den Bent, Ahmed Idbaih, Wolfgang Wick, Martin J. B. Taphoorn, Linda Dirven, Andrew Bottomley, Jaap C. Reijneveld, The EORTC Quality of Life Group and EORTC Brain Tumor Group

**Affiliations:** 1 Department of Medical Psychology, Amsterdam UMC, Vrije Universiteit Amsterdam, de Boelelaan 1118, PK 1Y 176, 1081 HZ Amsterdam, The Netherlands; 2 Brain Tumor Center Amsterdam, Amsterdam UMC, Vrije Universiteit Amsterdam, Amsterdam, The Netherlands; 3 Brain Tumor Center at Erasmus MC Cancer Institute, Rotterdam, The Netherlands; 4 Sorbonne Université, Inserm, CNRS, UMR S 1127, Institut du Cerveau et de la Moelle Épinière, ICM, AP-HP, Hôpitaux Universitaires La Pitié Salpêtrière - Charles Foix, Service de Neurologie 2-Mazarin, 75013 Paris, France; 5 Clinical Cooperation Unit Neurooncology, German Cancer Consortium (DKTK), German Cancer Research Center (DKFZ) & Department of Neurology, Heidelberg University Hospital, Heidelberg, Germany; 6 Department of Neurology, Leiden University Medical Centre, Leiden, The Netherlands; 7 Haaglanden Medical Centre, The Hague, The Netherlands; 8 Quality of Life Department, European Organization for Research and Treatment of Cancer, Brussels, Belgium; 9 Department of Neurology, Amsterdam UMC, Vrije Universiteit Amsterdam, Amsterdam, The Netherlands; 10 Department of Neurology, Stichting Epilepsie Instellingen Nederland (SEIN), Heemstede, The Netherlands

**Retraction note to: Quality of Life Research (2022) 31:3253–3266** 10.1007/s11136-022-03197-w

This article has been retracted at the request of the authors. An honest error occurred while exporting data from the original EORTC dataset which was stored as SAS and then transformed into Excel and from Excel into SPSS, significantly affecting the results and conclusions. A revised manuscript, reporting the correct data, will be submitted to the journal where it will be subject to conventional peer review. We would like to apologize to the scientific community, the editors, and the reviewers for this mistake. Ivan Caramanna, Martin Klein, Martin van den Bent, Ahmed Idbaih, Wolfgang Wick, Martin J. B. Taphoorn, Linda Dirven and Jaap C. Reijneveld agree to this retraction. Andrew Bottomley did not respond to correspondence from the Editor about this retraction.

